# Standardised Outcomes in Nephrology—Children and Adolescents (SONG-Kids): a protocol for establishing a core outcome set for children with chronic kidney disease

**DOI:** 10.1186/s13063-016-1528-5

**Published:** 2016-08-12

**Authors:** Allison Tong, Susan Samuel, Michael Zappitelli, Allison Dart, Susan Furth, Allison Eddy, Jaap Groothoff, Nicholas J. A. Webb, Hui-Kim Yap, Detlef Bockenhauer, Aditi Sinha, Stephen I. Alexander, Stuart L. Goldstein, Debbie S. Gipson, Camilla S. Hanson, Nicole Evangelidis, Sally Crowe, Tess Harris, Brenda R. Hemmelgarn, Braden Manns, John Gill, Peter Tugwell, Wim Van Biesen, David C. Wheeler, Wolfgang C. Winkelmayer, Jonathan C. Craig, on behalf of the SONG-Kids Investigators

**Affiliations:** 1 Sydney School of Public Health, The University of Sydney, Sydney, NSW Australia; 2 Centre for Kidney Research, The Children’s Hospital at Westmead, Westmead, NSW 2145 Australia; 3 Department of Pediatrics, Section of Nephrology, University of Calgary, Calgary, AB Canada; 4 Department of Pediatrics, Division of Pediatric Nephrology, Montreal Children’s Hospital, McGill University Health Centre, McGill University, Montreal, QC Canada; 5 Department of Pediatrics and Child Health, The Children’s Hospital Research Institute of Manitoba, University of Manitoba, Winnipeg, MB Canada; 6 Departments of Pediatrics and Epidemiology, Perelman School of Medicine and Division of Nephrology, The Children’s Hospital of Philadelphia, Philadelphia, PA USA; 7 Department of Pediatrics, University of British Columbia, Vancouver, BC Canada; 8 Department of Pediatric Nephrology, Emma Children’s Hospital AMC Academic Medical Center, Amsterdam, The Netherlands; 9 Department of Paediatric Nephrology and NIHR/Wellcome Trust Clinical Research Facility, University of Manchester, Manchester Academic Health Science Centre, Royal Manchester Children’s Hospital, Manchester, UK; 10 Department of Pediatrics, Yong Loo Lin School of Medicine, National University of Singapore, Singapore, Singapore; 11 UCL Centre for Nephrology and Great Ormond Street Hospital for Children NHS Foundation Trus, London, UK; 12 Division of Nephrology, Department of Pediatrics, All India Institute of Medical Sciences, New Delhi, India; 13 Division of Nephrology and Hypertension, Cincinnati Children’s Hospital Medical Center, Cincinnati, OH USA; 14 Department of Pediatrics, School of Medicine, University of Michigan, Ann Arbor, MI USA; 15 Crowe Associates Ltd, London, UK; 16 PKD International, London, UK; 17 Departments of Medicine and Community Health Sciences, Libin Cardiovascular Institute and O’Brien Institute of Public Health, University of Calgary, Calgary, AB Canada; 18 Division of Nephrology, University of British Columbia, Vancouver, BC Canada; 19 Department of Medicine, University of Ottawa, Ottawa, ON Canada; 20 Renal Division, Ghent University Hospital, Ghent, Belgium; 21 Centre for Nephrology, University College London, London, UK; 22 Selzman Institute for Kidney Health, Section of Nephrology, Baylor College of Medicine, Houston, TX USA

**Keywords:** Core outcome set, Outcomes research, Patient-centred outcomes, Clinical trials, Dialysis, Haemodialysis, Chronic kidney disease, Paediatrics

## Abstract

**Background:**

Children with chronic kidney disease (CKD), requiring dialysis or kidney transplantation, have a mortality rate of up to 30-fold higher than the general aged-matched population, and severely impaired quality of life. Symptoms such as fatigue and pain are prevalent and debilitating. Children with CKD are at risk of cognitive impairment, and poorer educational, vocational, and psychosocial outcomes compared with their well peers, which have consequences through to adulthood. Treatment regimens for children with CKD are long-term, complex, and highly intrusive. While many trials have been conducted to improve outcomes in children with CKD, the outcomes measured and reported are often not relevant to patients and clinicians, and are highly variable. These problems can diminish the value of trials as a means to improve the lives of children with CKD. The Standardised Outcomes in Nephrology—Children and Adolescents (SONG-Kids) study aims to develop a core outcome set for trials in children and adolescents with any stage of CKD that is based on the shared priorities of all stakeholders.

**Methods/Design:**

SONG-Kids involves five phases: *a systematic review* to identify outcomes (both domains and measures) that have been reported in randomised controlled trials involving children aged up to 21 years with CKD; *focus groups* (using nominal group technique) with adolescent patients and caregivers of paediatric patients (all ages) to identify outcomes that are relevant and important to patients and their family and the reasons for their choices; *semistructured key informant interviews* with health professionals involved in the care of children with CKD to ascertain their views on establishing core outcomes in paediatric nephrology; an *international three-round online Delphi survey* with patients, caregivers, clinicians, researchers, policy-makers, and members from industry to develop consensus on important outcome domains; and a *stakeholder workshop* to review and finalise the set of core outcome domains for trials in children with CKD (including nondialysis-dependent, dialysis, and kidney transplantation).

**Discussion:**

Establishing a core outcome set to be reported in all trials conducted in children with any stage of CKD will enhance the relevance, transparency, and impact of research to improve the lives of children and adolescents with CKD.

## Background

Children with chronic kidney disease (CKD) requiring dialysis or kidney transplantation have a mortality rate of up to 30-fold higher than the general population [1]. CKD also severely impacts the quality of life of affected children and their caregivers [2–7]. Symptoms such as fatigue, pain, and oedema, are prevalent and debilitating [8, 9]. Patients and their caregivers are required to manage a complex and onerous treatment regimen involving polypharmacy, diet and fluid restrictions, ongoing clinical appointments, and for children dependent on renal replacement therapy, a highly technical and invasive dialysis regimen or kidney transplantation; all while endeavouring to achieve growth and developmental milestones [5, 10]. Children with CKD are at an increased risk of low educational attainment, cognitive impairment, and worse vocational, psychological, social, and behavioural outcomes compared with their well peers [6, 7, 11–14], and these have consequences through to adulthood [15, 16].

Clinical trials have been conducted in children with CKD in an effort to improve such unacceptably poor outcomes. However, the potential of trials to improve outcomes can only be realised if they address the problems of relevance to children and families affected by CKD, and measure the outcomes that are valued by stakeholders, including children, caregivers and parents, clinicians and policy-makers. Unfortunately, empiric studies in other specialties, have shown that trials commonly use surrogate endpoints that may not be directly meaningful to patients and clinicians, and a plethora of different outcome measures [17]. This can limit the utility of trials in generating relevant and reliable evidence for decision-making, which contributes to ‘research waste’ [17].

Surrogate outcomes, such as haematological, biochemical, or imaging outcomes, are commonly used in trials as they require a smaller sample size, a shorter duration, and fewer resources to assess the efficacy of treatment; however, they are largely nonvalidated, heterogeneous, and may not be relevant to patients. In the Cochrane Central Register of Controlled Trials [18], trials in children with CKD appear to be largely focussed on growth, anaemia, nutrition, immunosuppression, and dialysis-related interventions and mostly report surrogate outcomes such as parathyroid hormone, serum calcium, and haemoglobin status, and kidney function [19–25]. Systematic reviews have shown that patient-relevant outcomes, such as mortality and cardiovascular disease, are infrequently included [25, 26] and quality of life outcomes are scarcely reported. Studies that have directly elicited perspectives from children with CKD have identified that anxiety, school attendance and achievement, social participation, hospitalisation, and fatigue, are important and relevant to them [7, 10, 27–29], yet these are not typically measured or reported in trials.

The uncertainty about the relevance of trial outcomes to patients, caregivers, and clinicians has important implications for children with CKD, as treatment can be highly intrusive and onerous, with profound impacts on health and lifestyle. Also, shared decision-making can be particularly challenging in this vulnerable population given their younger age and higher risk of neurocognitive dysfunction [12, 13, 30]. These challenges highlight the need for systematic, collaborative and concerted efforts to engage children with CKD and their families in explicitly identifying the outcomes that they regard as important for research, and to understand the reasons for their preferences.

Systematic problems are also evident in how outcomes are measured and reported. Substantial variability is usually found, making judgements about the relative efficacy of interventions very difficult, and this contributes to inefficiencies in research. For example, in a Cochrane review of recombinant human growth hormone for children in any stage of CKD involving 16 trials (*n* = 809 children), 7 (44 %) trials reported change in height standard deviation score (SDS), 6 (38 %) reported height velocity, 3 (17 %) reported height SDS, 3 (17 %) reported height velocity SDS, and 2 (11 %) reported change in height velocity.

Many initiatives have been established worldwide to develop core outcome sets to improve the relevance and consistency of outcome measurement and selection. Core outcomes are defined as the minimum set of outcomes that should be measured and reported in clinical trials of a specific condition, though may also be used for other types of research and quality improvement activities [31]. Standard sets of outcomes have also been touted as ‘a practical and decisive step in accelerating value improvement in health care’ [32]. The Outcome Measures in Rheumatology (OMERACT) commenced in 1992 and set foundational work in the development of core outcomes, which has led to more complete reporting of relevant outcomes in rheumatology trials. The Core Outcome Measures in Effectiveness Trials (COMET) was then formed to provide a central resource and portal for the development and dissemination of core outcome sets [31]. Consensus-based core outcome sets for paediatric conditions are sparse, but have been conducted in the areas of otitis media [33, 34], asthma [35], neurodisability [36], autism [37], and cerebral palsy [38]. Recently, there have been major efforts to validate patient-reported outcome measures in children with CKD to assess disease severity and activity [39]; however, the outcome domains that are explicitly prioritised by patients and clinicians to be important remain to be identified. No core outcome set exists in paediatric nephrology.

The Standardised Outcomes in Nephrology—Children and Adolescents (SONG-Kids) study aims to establish a core outcome set for trials in children and adolescents aged up to 21 years of age across all stages of CKD (including nondialysis-dependent, dialysis, kidney transplantation), which may also be used in other research contexts. The upper age limit to define the paediatric population varies internationally and is up to 21 years in the United States [40]. The SONG-Kids Steering Group was convened in February 2016, and is comprised of 14 paediatric nephrologists from seven countries who have experience in clinical trials, outcomes research, and implementation. The specific objectives of SONG-Kids are to: (1) describe the scope and consistency of outcomes used in haemodialysis trials, (2) identify outcomes that are important to children with CKD and their caregivers and elucidate the reasons for their choices, (3) ascertain health professional perspectives on core outcomes in paediatric CKD, (4) develop a core outcome domain set based on consensus across all stakeholder groups including patients/caregivers and health professionals, and (5) establish a core outcome domain set for trials in children with CKD.

## Methods/design

**Fig. 1:**
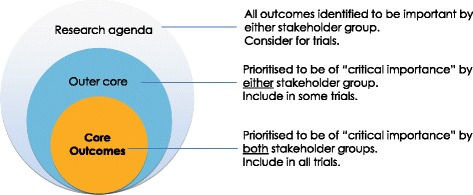
SONG-Kids process

The SONG-Kids methodology is adapted from the processes used in the SONG initiative [41] and the World Health Organisation (WHO)-endorsed OMERACT framework [42] as these have been recognised as a valid approach for establishing core outcomes. SONG-Kids involves five phases: systematic review, focus groups with nominal group technique conducted with adolescents with CKD and their parents (or other caregivers), stakeholder interviews, an online Delphi survey, and a consensus workshop (Fig. 1).

### Phase 1: systematic review of outcome domains and outcome measures reported in trials of interventions for children with chronic kidney disease

We will conduct a systematic review to identify and compare outcome domains and measures reported in randomised controlled trials (RCTs) of interventions for children in any stage of CKD.

#### Search strategy

We will search the Cochrane Central Register of Controlled Trials (CENTRAL) to find all RCTs involving children with any CKD diagnosis and treatment stage of CKD (CKD stages 1–5 (nondialysis-dependent), 5D (on haemodialysis or peritoneal dialysis), and 5 T (kidney transplantation)). No date or language restrictions will be applied.

#### Types of studies

We will include all RCTs published in peer-reviewed journals. Conference reports and abstracts will not be included as we are assessing the outcome reported, not the results associated with those outcomes. Given the space constraints of abstracts and conference reports, they are not a reliable source of all of the outcomes measured and reported in trials.

#### Types of interventions

Any intervention for children with CKD (CKD stages 1–5 (nondialysis-dependent), CKD stage 5D (haemodialysis or peritoneal dialysis), or CKD stage 5 T (kidney transplantation)) will be eligible. Types of interventions can include, but will not be limited to, pharmacological, surgical, psychosocial, and health service interventions.

#### Types of participants

Children aged 21 years or below with CKD. A trial will be eligible if it can be determined that more than 50 % of participants across all arms are aged 21 years or below.

#### Exclusion criteria

RCTs that only include patients aged above 21 years, or include children with chronic conditions in which the data from the CKD population are not reported separately, will be excluded.

#### Eligibility of studies

Two reviewers will independently assess all records obtained from the searches. Full texts of all potentially relevant systematic reviews and RCTs will be assessed independently by the two reviewers and any disagreement on the eligibility of included studies will be resolved through consultation with a third reviewer.

#### Data extraction

The primary reviewer will extract from all included trials the following: first author, date of publication, country/ies in which the trial was conducted, sample size, participant characteristics (age, gender), trial duration, treatment stage of CKD (nondialysis-dependent CKD, haemodialysis, peritoneal dialysis, living donor and deceased donor kidney transplantation), name and type of intervention (e.g. pharmacological, surgical, psychosocial, lifestyle), and all outcomes as reported in the trial (definitions, specific measures, thresholds, time points or time frames for measurement, change in level or percentage, scores). At least three reviewers (including two paediatric nephrologists) will cross-check the data extraction.

#### Data analysis and presentation

The data will be entered into Microsoft Excel to assist with data management. Two reviewers will group similar outcomes into outcome domains, and the outcome domains will then be classified as surrogate (biochemical or physiological outcomes that may or may not be validated), clinical (medical outcome based on clinician assessment), or patient-reported outcomes (outcomes reported by the patients usually relating to quality of life or symptoms). These classifications will be reviewed and discussed by the SONG-Kids Steering Group via email and teleconference. Any disagreements will be resolved by consensus among the Steering Group. We will ascertain the frequency with which each outcome domain was reported across trials. For each outcome domain, we will count the number of different outcomes (including measures) and the number of trials that assessed each specific outcome. We will use the software package R (version 3.2.3) to perform all statistical analyses.

### Phase 2: focus groups with modified nominal group technique

Children and parents of children with CKD will be asked to identify and prioritise outcomes that they consider are relevant and important to measure in trials, and to discuss the reasons for their choices. The nominal group technique is a transparent and systematic process for achieving consensus within a structured group discussion, and has been used to define research and health service priorities [43–47]. This technique is useful for generating a prioritised set of ideas and minimises individual participants from dominating the discussion, facilitates equitable and active contribution, and mitigates direct criticism and rejection among participants [47]. Previous studies have demonstrated the feasibility of focus groups with prioritisation exercises in children [48–50].

#### Participants and recruitment

Children aged 12 to 21 years with CKD (not on dialysis (CKD stages 1–5)), on haemodialysis or peritoneal dialysis (CKD stage 5D), or who have received a kidney transplantation (CKD stage 5 T)) and parents/caregivers of children aged 21 years or below with CKD are eligible to participate. Recruitment will be mostly focussed on children receiving treatment for CKD who are likely to progress to end-stage kidney disease, and who are able to discuss their experiences and perspectives of CKD in the English language. Most children with early stage CKD are asymptomatic and may not be receiving treatment, thus we will focus on recruiting children with more advanced CKD or those who are receiving renal replacement therapy in the form of dialysis or kidney transplantation. All causes and types of CKD (e.g. birth defects, hereditary diseases, hereditary nephritis, infection, nephrotic syndrome, systemic diseases (lupus nephritis, diabetes)) will be included.

We will convene a total minimum of 16 focus groups (with ten participants per group) based on the following population:

1. Children aged 12 to 21 years not on renal replacement therapy (four groups)
2. Children aged 12 to 21 years on renal replacement therapy in the form of dialysis of kidney transplantation (four groups)
3. Parents of children (aged 0 to 21 years) not on renal placement therapy (four groups), and
4. Parents of children (aged 0 to 21 years) on renal replacement therapy (four groups)

The groups will be convened until data saturation, i.e. when no new outcomes or perspectives relevant to the study are being raised in subsequent groups. Depending on the patient population at each participating site, we will convene the child focus groups by treatment and age group (12–16 years and 17–21 years) if feasible.

Participants will be recruited from the participating centres in Australia (The Children’s Hospital at Westmead, Royal Children’s Hospital, Royal Children’s and Mater Children’s Hospital), Canada (Alberta Children’s Hospital, BC Children’s Hospital), and the United States (Baylor College of Medicine). A purposive sampling will be applied to include a broad range of demographic (age, gender, socioeconomic status, educational level, ethnicity) and clinical (patient stage of CKD including treatment modality, cause or diagnosis of CKD, comorbidities) characteristics. Informed consent will be obtained from all participants aged 18 years and over. Participants under 18 years of age (or otherwise depending on local regulations) will be asked to provide written consent or assent; and consent will be sought from their adult legal guardian in accordance with ethical requirements at each participating site.

#### Data collection

The focus groups will be up to 2 hours in duration and convened in a meeting room external to the hospital setting to encourage open discussion in a neutral environment. The questions will be adapted from focus group guides used to elicit perspectives on outcomes for research among adult patients who are on haemodialysis or have received a kidney transplantation [44, 46], and discussed among the Steering Group. A facilitator with training and experience in nominal group technique will moderate the discussion. A note-taker will record notes on the group dynamics, disposition and interaction among the participants. Separate focus-group-run sheets and timings will be developed for parents and children; however, both sets of focus groups’ questions will cover the three stages described in the following section:

1. Welcome and introduction, including an explanation about research and outcomes (15 min)
2. Focus group discussion (20 min): participants will be asked to talk generally about their experiences and perspectives of CKD, aspects of CKD or treatment that are most important to participants, the perceived benefit, harms and challenges of different treatment/interventions
3. Nominal group technique (50 min): participants will be asked to write one or two outcomes on a notepad that they think are important for research. The facilitator (AT/CSH) will ask participants in turn to read out their suggestions, and will write (draw) them on a board. Each outcome will be discussed and defined. Other outcomes identified from phase 1 (systematic review) will be added to the list. A list will be printed for participants. Participants will be asked to rank the outcomes (at least the top ten) in order of importance from 1 (most important) to X (least important).

Participants will be given the opportunity to discuss any similarities and differences in their opinion. All focus groups will be audio-recorded and transcribed verbatim. The outcomes from each group will be reviewed and discussed among the investigator team, which include paediatric nephrologists.

#### Data analysis

Quantitative rating/ranking: we will extract the top ten ranked outcomes for each participant. The highest ranked outcome for each participant will be reverse-coded and assigned a value of 10, and the least important a value of 1. Outcomes that were excluded from the top ten or not ranked will be given a value of zero. The individual rank scores for all participants across all groups will be used to determine the mean rank score for the top ten most important outcomes from the full set of outcomes. We will also assess the proportion of participants who ranked a specific outcome in the top ten most important outcomes. This will be used to generate a list of outcomes ordered by the mean priority score. We will also calculate the mean rank scores separately for patients and parents/caregivers with the statistical significance of the differences assessed on the basis of a *t* test, considered significant at *p* < 0.05. A covariate adjustment for group composition will also be conducted. The analysis will be conducted using the statistical package SPSS.

Qualitative analysis: the transcripts will be imported into HyperRESEARCH (ResearchWare Inc. www.researchware.com, version 3.7.2) software for qualitative data analysis. Using thematic analysis, we will review the transcripts line-by-line and inductively code for concepts and themes that emerge from the data that provide the reasons underpinning the participants’ ranking choices. Similar concepts will be grouped into themes and subthemes. The preliminary findings will be discussed among the research team to ensure that the analytical themes capture the diversity and depth of the data.

### Phase 3: semistructured key informant interviews

Semistructured key informant interviews will be conducted with health professionals to ascertain a range and depth of their individual perspectives on establishing core outcomes for research in children with CKD. We will follow the Consolidated Criteria for Reporting Qualitative Health Research (COREQ) [51].

#### Participants and recruitment

Health professionals who have experience and expertise in paediatric CKD will be eligible to participate in an interview. Health professionals will include paediatric nephrologists, surgeons, nurses, allied health professionals (e.g. psychologists, social workers, dieticians), researchers, and policy-makers. A minimum of 50 participants will be recruited across all regions worldwide and identified from established networks of the Steering Group and Investigators. Participants will be purposively sampled to ensure that a range of demographic and professional roles and experiences are obtained. We will recruit until data saturation, which we will define as when no new concepts or outcomes are being identified in three consecutive interviews. We will obtain informed consent from all participants.

#### Data collection

The interview guide will incorporate findings from phases 1 and 2. The interviews will be conducted face-to-face; however, Skype or telephone interviews will be an option if preferred by the participants or when an in-person interview cannot be feasibly arranged. Participants will be prompted to discuss their perspectives on: (1) their experiences in providing care for children with CKD and aspects of treatment that are important and challenging, (2) outcomes currently measured and reported in paediatric nephrology trials and the implications on practice and policy, (3) outcomes they believe are important and relevant for trials and the reasons why, and (4) perspectives on the development and implementation of core outcomes for trials in children with CKD. The interview will take approximately 40 min, and will be audio-recorded and transcribed.

#### Data analysis

From the transcripts, we will extract all outcomes identified by participants (to inform the Delphi survey in phase 4). We will use thematic analysis, as described in phase 2, to identify the themes that reflect their perspectives, beliefs, priorities, and values about core outcomes for research in children with CKD. To ensure that the full range and depth of the data are included in the analysis, at least two investigators (AT, CSH), will be involved in coding the data analysis to develop descriptive and analytical themes (investigator triangulation). The preliminary results will also be reviewed by the interview participants (member checking) and the SONG-Kids Steering Group.

### Phase 4: international online Delphi consensus survey

An international online SONG-Kids Delphi survey will be conducted to generate consensus on the outcome domains that are most important to all stakeholder groups. The Delphi survey has been used as a reliable approach for gaining consensus on core outcome sets across a range of health conditions [34, 52–57]. This technique involves two or three rounds of surveys completed anonymously by participants who have knowledge, experience, or expertise on the topic [47, 58]; this allows participants to contribute their perspectives and provide feedback on the group results, and allows respondents to revise their opinions after reflecting on the group responses [59]. This process facilitates equitable contribution from all members of the Delphi panel as participants are protected from direct confrontation so they are able to communicate their individual perspectives. We will use a reporting checklist for Delphi studies on developed core outcomes, developed by Sinha et al. (2011), which addresses the size and composition of the Delphi panel, methodology and process, and results of the Delphi survey [58]. However, the approach will be adapted for the child version of the Delphi survey for age-appropriateness. The Delphi survey has been used in children with chronic conditions [27, 34, 35, 38].

#### Participants and recruitment

There is no standard sample size calculation or recommendation for a Delphi study [60]. Most Delphi surveys used to develop core outcomes have included fewer than 200 respondents, though a Delphi survey used to develop core outcomes for trials involving adults on haemodialysis (SONG-HD) and kidney transplantation (SONG-Tx) included approximately 1000 participants.

Our minimum target sample size will be 500 respondents with at least 100 children aged 8–21 years with CKD (not on dialysis (CKD stages 1–5), on haemodialysis or peritoneal dialysis (CKD stage 5D), or who have received a kidney transplantation (CKD stage 5 T)); 150 caregivers of children with CKD, and 250 health professionals. This is based on the estimated patient population at recruiting hospitals, and members of health professional societies in paediatric nephrology. We will aim to recruit physicians (paediatric nephrologists, surgeons, psychiatrists (minimum *n =* 170); nursing and allied health professionals (pharmacists, dietitians, psychologists, social workers) (*n* = 50); and policy-makers, researchers, and representatives from industry (*n* = 30); who have experience, expertise, or interest in outcomes in children with CKD.

We will seek to obtain a broad range of demographics, clinical characteristics (patients), and professional experiences and roles (health professionals); and will also use snowballing strategies (where participants can nominate or extend an invitation to other relevant stakeholder members to participate). Patients/caregivers will be primarily recruited via the investigator’s hospital/university sites, which are in Australia, Canada, Europe, the United Kingdom, the United States, and Singapore, and patient/consumer organisations, and the SONG Initiative database. Health professionals will be invited via professional paediatric nephrology societies and existing networks of the SONG-Kids Steering Group and Investigators.

#### Data collection

##### Generating the list of outcomes

The Delphi survey will include outcome domains from the systematic review of outcomes reported in RCTs (phase 1) and outcomes identified in the nominal group technique study (phase 2).

All outcomes will include a plain language definition (Flesch-Kincaid Index of grade 5 readability (age 10 years)). The child version for patients aged 8 to 18 years will include a picture to illustrate the outcome and an explanation in child-friendly terms. An audio button will also be embedded in each outcome so participants can hear the outcome read aloud. The adult version for patients aged 18 to 21 years, caregivers, and health professionals will also include a more detailed definition if appropriate. The survey will be reviewed by the SONG Executive and SONG-Kids Steering Group and Investigators, which includes members who are children with CKD and parents of children with CKD, and will be piloted with at least five patients with CKD and five parents/caregivers.

##### Survey administration

All participants will register their name and email address via www.songinitiative.org after receiving an information and consent form from recruiting sites. Participants recruited via collegial networks or social media will receive an information form upon registration. A statement of informed consent will be provided at the beginning of the survey. For children aged under 18 years, parents will also be required to provide consent for their child to participate. The surveys will be administered using the online survey platform, LimeSurvey, which will be optimised for viewing using both desktop and smartphone. Each participant will be given a unique identifier so we can monitor and link their responses anonymously. At least three reminders will be sent to participants during the Delphi survey rounds in an attempt to retain at least a 70 % response rate across all three rounds. Participants who complete all three rounds will receive a copy of the preliminary results to provide feedback and comment.

##### Delphi survey round 1

Participants will be presented with the question: ‘how important is this outcome for research in children with CKD?’ and asked to rate the importance of each outcome domain (approximately 30) using the GRADE 9-point Likert scale [61]. The visual scale used for each outcome will indicate ratings 1 to 3 as of ‘limited importance’, 4 to 6 as ‘important, but not critical’, and 7 to 9 as of ‘critical importance’. An option ‘I don’t know’ will also be provided.

For each outcome, a free-text box will be provided so participants can provide optional comments. To minimise ordering bias, the order of outcomes shown will be randomised. Participants can suggest new outcomes. All new outcomes that are suggested by more than 10 % of participants that do not overlap or duplicate outcomes will be recoded by at least two investigators (NE, CSH) and reviewed by the SONG-Kids Steering Group, then carried through to round 2.

**Fig. 2:**
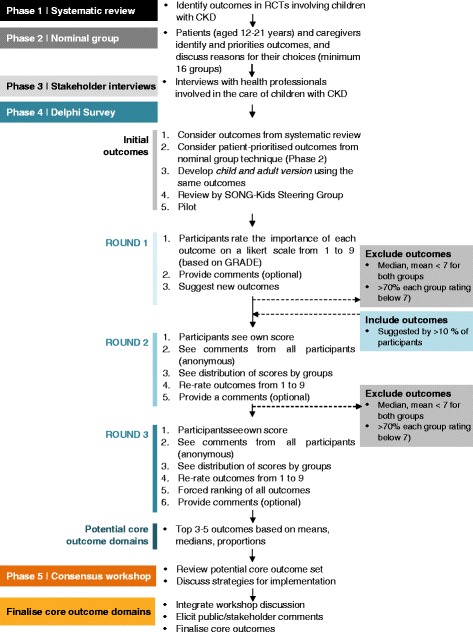
Conceptual schema of core outcomes

We will review the distribution of scores across all outcomes. Any outcome with a median and mean of more than 7 (with 70 % or more participants in both patient/caregiver and health professionals rating the outcome 7–9; based on the OMERACT criteria for "consensus in") will be carried through to round 2. Any outcomes excluded in subsequent rounds will be listed in the research agenda (Fig. 2).

##### Delphi survey round 2

In round 2, participants will be presented with a column graph of the distribution of scores for each outcome for the following groups: (1) patients/family members/donors, (2) health professionals, and (3) all participants (with scores weighted evenly between groups). An explanation of how to read the graph will be provided to ensure that participants can understand and interpret the graph clearly. For the child version, an animation to explain how to understand the group results will be shown. They will also see comments from round 1 by patients/family members/donors and health professionals. The comments may be edited for clarity and readability. Their own response from the previous round will be highlighted in the rating scale. Participants will rerate each outcome and any additional outcomes identified in round 1 using the same 9-point Likert scale. An optional free-text box will be provided for participants to state reasons for their rating, or to provide feedback or responses to the participant comments.

All outcomes with a median and mean of more than 7 (with 70 % or more participants in both patients/caregivers and health professionals rating the outcome 7–9) will be included in round 3.

##### Delphi survey round 3

Participants will see the distribution of scores for each outcome for all participants and by stakeholder groups, and comments from round 2 in the same format as the previous round. Participants will see their own score from round 2 highlighted in the rating scale and rerate all outcomes. A free-text box will be provided for participants to make any additional comments. After the rating questions, participants will be asked to complete a forced ranking exercise. They will be presented with a list of all outcomes to arrange into a list in order of importance from most important to least important.

#### Data analysis

For all three rounds, we will summarise the distribution of scores and calculate the mean, median and proportion for ratings and rankings of each outcome. The OMERACT prespecified definition of ‘consensus’ for outcomes to be included in the core set states that the outcome must have at least 70 % of participants scoring 7 to 9, and fewer than 15 % of participants scoring as 1 to 3 [41]. Based on previous initiatives and feasibility, three to five outcomes will be included in the core outcome set. If more than five outcomes will meet the OMERACT threshold for inclusion, the preliminary core outcome domains will also be determined based on means, medians, and proportions in round 3, which will be validated against the OMERACT cut-offs based on proportions. Since the thresholds may need to be defined post hoc, the rationale and threshold for inclusion will be detailed in a plain language report for transparency. This will also be discussed in the consensus workshop outlined in the next section.

### Phase 4: Consensus workshop

A consensus workshop will be convened with stakeholders to review the results from phases 1 to 3 and discuss the identification of core outcomes (including the potential set of core outcomes). The workshop will be held in conjunction with an international paediatric nephrology conference. SONG-Kids Steering Group members will chair and facilitate the session. Approximately 60 delegates; including at least 20 patients/family members will be invited to attend the workshop. Health professionals with a range of clinical experience in paediatric nephrology (nephrologists, surgeons, nursing and allied health professionals), expertise in research (epidemiology, clinical trials in children with CKD, registries, quality improvement), and leadership or advisory roles in major research and policy organisations (including regulators), and industry will be invited to attend.

Prior to the workshop, we will send participants a copy of the results from phases 1 to 3. The child version will include a link to an online animation to explain the results, and all materials will be developed to meet the Flesch-Kincaid Index of grade 5 readability (age 10 years). Participants will be asked to reflect on the results to date so they may be better prepared to contribute their feedback during the workshop. The workshop will include three sessions:

- Session 1: Introduction
  We will provide a brief introduction to the SONG-Kids initiative and present the details of the SONG-Kids process and results from phases 1 to 3, and the preliminary core outcome set and proposed threshold for inclusion.
- Session 2: Breakout groups
  Participants will be allocated to five breakout groups with up to 12 participants in each group (including a facilitator and a cofacilitator). Mixed stakeholder groups with at least two family members will be convened to encourage a richer exchange of ideas, explanations of similar or different opinions, and breadth of discussion. Patients aged 12 to 18 years will be allocated to a separate group.
  A trained facilitator will ask participants to discuss the identification and implementation of core outcomes, and ensure cooperative, respectful, and inclusive discussion. All facilitators will attend a briefing session and will be provided with a question guide.
- Session 3: Plenary discussion
  The groups will reconvene to engage in a broader discussion moderated by the workshop chair. One person from each breakout group will present a brief summary of their discussion. The wider group will be invited to give their opinions and reflections on the issues raised by other groups. The moderator will summarise key similarities and differences in the points raised across groups.

The moderator may ask participants to formally endorse the proposed core outcomes’ set.

### Finalisation of core outcome domains

The SONG-Kids process (phases 1 to 4) and proposed outcomes will be published in a plain language report for circulation to the participants in the Delphi (phase 3) and consensus workshop (phase 4), circulated to stakeholder groups and made available on the website for 3 weeks to obtain public comment. A child version will also be developed. All feedback will be reviewed by the SONG-Kids Steering Group and SONG Executive Committee to finalise the SONG-Kids core outcome set.

## Discussion

The SONG-Kids project uses a systematic and transparent process that will engage children, caregivers, clinicians, policy-makers, and researchers to develop a prioritised set of core outcome domains for trials in children with CKD based on consensus. The core outcomes will help to ensure that the outcomes of real importance to children, their families, and health care professionals involved in their care, are the focus of attention—not necessarily that which is easy to measure, or has been measured and reported historically. The core outcomes may also be considered for other types of research including observational studies and quality improvement projects. Once the core outcome domains are finalised we will identify, and if necessary, develop core outcome measures that are valid, discriminative and feasible.

We will consult with key stakeholders to ensure that the core outcomes will be disseminated and translated via international and national policy and research organisations. Complete and consistent reporting of outcomes that are highly relevant to patients, practitioners, policy-makers, and researchers will improve the relevance and value of research for informing decision-making in the care of children with CKD. Ultimately, this may also direct the research agenda towards improving outcomes that are important to patients, providers, and policy-makers.

### Study status

Recruitment and data collection have commenced.

## Abbreviations

CKD: Chronic Kidney Disease
COMET: Core Outcome Measures Effectiveness Trials
OMERACT: Outcome Measures Rheumatoid Arthritis Clinical Trials
RCT: Randomised Controlled Trial
SONG-Kids: Standardised Outcomes in Nephrology-Children and Adolescents
WHO: World Health Organisation

## References

[1] McDonald SP, Craig JC (2004). Long-term survival of children with end-stage renal disease. N Eng J Med.

[2] Moreira JM, Bouissou Morais Soares CM, Teixeira AL, Simões E Silva AC, Kummer AM (2015). Anxiety, depression, resilience and quality of life in children and adolescents with pre-dialysis chronic kidney disease. Pediatr Nephrol.

[3] Lopes M, Ferraro A, Koch VH (2014). Health-related quality of life of children and adolescents with CKD stages 4-5 and their caregivers. Pediatr Nephrol.

[4] Tong A, Wong G, McTaggart S, Henning P, Mackie F, Carroll RP, Howard K, Craig JC (2013). Quality of life of young adults and adolescents with chronic kidney disease. J Pediatr.

[5] Tong A, Lowe A, Sainsbury P, Craig JC (2008). Experiences of parents who have children with chronic kidney disease: a systematic review of qualitative studies. Pediatr.

[6] Gerson AC, Wentz A, Abraham AG, Mendley SR, Hooper SR, Butler RW, Gipson DS, Lande MB, Shinnar S, Moxey-Mims MM (2010). Health-related quality of life of children with mild to moderate chronic kidney disease. Pediatr.

[7] Tong A, Tjaden L, Howard K, Wong G, Morton R, Craig JC (2011). Quality of life of adolescent kidney transplant recipients. J Pediatr.

[8] Davis ID, Greenbaum LA, Gipson D, Wu LL, Sinha R, Matsuda-Abedini M, Emancipator JL, Lane JC, Hodgkins K, Nailescu C (2012). Prevalence of sleep disturbances in children and adolescents with chronic kidney disease. Pediatr Nephrol.

[9] Roumelioti ME, Wentz A, Schneider MF, Gerson AC, Hooper S, Benfield M, Warady BA, Furth SL, Unruh ML (2010). Sleep and fatigue symptoms in children and adolescents with CKD: a cross-sectional analysis from the chronic kidney disease in children (CKiD) study. Am J Kidney Dis.

[10] Tong A, Henning P, Wong G, McTaggart S, Mackie F, Carroll RP, Craig JC (2013). Experiences and perspectives of adolescents and young adults with advanced CKD. Am J Kidney Dis.

[11] Lande MB, Gerson AC, Hooper SR, Cox C, Matheson M, Mendley SR, Gipson DS, Wong C, Warady BA, Furth SL (2011). Casual blood pressure and neurocognitive function in children with chronic kidney disease: a report of the children with chronic kidney disease cohort study. Clin J Am Soc Nephrol.

[12] Haavisto A, Korkman M, Holmberg C, Jalanko H, Qvist E (2012). Neuropsychological profile of children with kidney transplants. Nephrol Dial Transplant.

[13] Hooper SR, Gerson AC, Johnson RJ, Mendley SR, Shinnar S, Lande MB, Matheson MB, Gipson DS, Morgenstern B, Warady BA, et al. Neurocognitive, social-behavioural, and adaptive functioning in preschool children with mild to moderate kidney disease. J Dev Behav Pediatr. 2016:Feb 17. [E-pub head of print]. 10.1097/DBP.0000000000000267 PMC4818179 26890559

[14] Thys K, Schwering KL, Siebelink M, Dobbels F, Borry P, Schotsmans P, Aujoulat I (2015). Psychosocial impact of pediatric living-donor kidney and liver transplantation on recipients, donors, and the family: a systematic review. Transplant Int.

[15] Tjaden LA, Vogelzang J, Jager KJ, van Stralen KJ, Maurice-Stam H, Grootenhuis MA, Groothoff JW (2014). Long-term quality of life and social outcome of childhood end-stage renal disease. J Pediatr.

[16] Lewis H, Mark SD (2014). Differences between paediatric and adult presentation of ESKD in attainment of adult social goals. Pediatr Nephrol.

[17] Chalmers I, Bracken MB, Djulbegovic B, Garattini S, Grant J, Gülmezoglu AM, Howells DW, Ioannidis JP, Oliver S (2014). How to increase value and reduce waste when research priorities are set. Lancet.

[18] Cochrane Central Register of Controlled Trials (CENTRAL). Available at http://onlinelibrary.wiley.com/cochranelibrary/search?searchRow.searchOptions.searchProducts=clinicalTrialsDoi. Accessed 11 Mar 2016.

[19] Bacchetta J, Wesseling-Perry K, Kuizon B, Pereira RC, Gales B, Wang HJ, Elashoff R, Salusky IB (2013). The skeletal consequences of growth hormone therapy in dialyzed children: a randomized trial. Clin J Am Soc Nephrol.

[20] Schmitt CP, Nau B, Gemulla G, Bonzel KE, Hölttä T, Testa S, Fischbach M, John U, Kemper MJ, Sander A (2013). Effect of the dialysis fluid buffer on peritoneal membrane function in children. Clin J Am Soc Nephrol.

[21] Goldstein SL, Morris D, Warady BA (2013). Comparison of the safety and efficacy of 3 iron sucrose iron maintenance regimens in children, adolescents, and young adults with CKD: a randomized controlled trial. Am J Kidney Dis.

[22] Hokken-Koelega A, Mulder P, De Jong R, Lilien M, Donckerwolcke R, Groothof J (2000). Long-term effects of growth hormone treatment on growth and puberty in patients with chronic renal insufficiency. Pediatr Nephrol.

[23] Hahn D, Hodson EM, Craig JC (2015). Interventions for metabolic bone disease in children with chronic kidney disease. Cochr Data Sys Rev.

[24] Hodson EM, Willis NS, Craig JC (2012). Growth hormone for children with chronic kidney disease. Cochrane Database Syst Rev.

[25] Chaturvedi S, Jones C (2007). Protein restriction for children with chronic kidney disease. Cochr Data Sys Rev.

[26] Albaramki J, Hodson EM, Craig JC, Webster AC (2012). Parenteral versus oral iron therapy for adults and children with chronic kidney disease. Cochrane Database Syst Rev.

[27] Sattoe JN, Hilberink SR, Peeters MA, van Staa A (2014). ‘Skills for growing up’: supporting autonomy in young people with kidney disease. J Ren Care.

[28] Nicholas DB, Picone G, Selkirk EK (2011). The lived experiences of children and adolescents with end-stage renal disease. Qual Health Res.

[29] Tjaden L, Tong A, Henning P, Groothoff J, Craig JC (2012). Children’s experiences of dialysis: a systematic review of qualitative studies. Arch Dis Child.

[30] Ruebner RL, Laney N, Kim JY, Hartung EA, Hooper SR, Radcliffe J, Furth SL (2016). Neurocognitive dysfunction in children, adolescents, and young adults with CKD. Am J Kidney Dis.

[31] Prinsen CA, Vohra S, Rose MR, King-Jones S, Ishaque S, Bhaloo Z, Adams D, Terwee CB (2014). Core Outcome Measures in Effectiveness Trials (COMET) initiative: protocol for an international Delphi study to achieve consensus on how to select outcome measurement instruments for outcomes included in a ‘core outcome set’. Trials.

[32] Porter ME, Larsson S, Lee TH (2016). Standardizing patient outcomes measurement. N Eng J Med.

[33] Harman NL, Bruce IA, Callery P, Tierney S, Sharif MO, O’Brien K, Williamson PR (2013). MOMENT—Management of Otitis Media with Effusion in Cleft Palate: protocol for a systematic review of the literature and identification of a core outcome set using a Delphi survey. Trials.

[34] Harman NL, Bruce IA, Kirkham JJ, Tierney S, Callery P, O’Brien K, Bennett AM, Chorbachi R, Hall PN, Harding-Bell A (2015). The importance of integration of stakeholder views in core outcome set development: otitis media with effusion in children with cleft palate. PLoS One.

[35] Sinha IP, Gallagher R, Williamson PR, Smyth RL (2012). Development of a core outcome set for clinical trials in childhood asthma: a survey of clinicians, parents, and young people. Trials.

[36] Morris C, Janssens A, Shilling V, Allard A, Fellowes A, Tomlinson R, Williams J, Thompson Coon J, Rogers M, Beresford B (2015). Meaningful health outcomes for paediatric neurodisability: stakeholder prioritisation and appropriateness of patient reported outcome measures. Health Qual Life Outcomes.

[37] McConachie H, Parr JR, Glod M, Hanratty J, Livingstone N, Oono IP, Robalino S, Baird G, Beresford B, Charman T (2015). Systematic review of tools to measure outcomes for young children with autism spectrum disorder. Health Technol Assess.

[38] Vargus-Adams JN, Martin LK (2009). Measuring what matters in cerebral palsy: a breadth of important domains and outcome measures. Arch Phys Med Rehabil.

[39] Selewski DT, Massengill SF, Troost JP, Wickman L, Messer KL, Herreshoff E, Bowers C, Ferris ME, Mahan JD, Greenbaum LA (2014). Gaining the patient reported outcomes measurement information system (PROMIS) perspective in chronic kidney diseases: a Midwest Pediatric Nephrology Consortium Study. Pediatr Nephrol.

[40] Williams K, Thomson D, Seto I, Contopoulos-Ioannidis DG, Ioannidis JP, Curtis S, Constantin E, Batmanabane G, Hartling L, Klassen T (2012). Standard 6: age groups for pediatric trials. Pediatr.

[41] Boers M, Kirwan JR, Tugwell P, Beaton D, Binghma CO, Conaghan PG, D’Agostino MA, de Wit M, Gossec L, March L (2014). The OMERACT Handbook.

[42] Stucki G, Boonen A, Tugwell P, Cieza A, Boers M (2007). The World Health Organisation International Classification of Functioning, Disability and Health: a conceptual model and interface for the OMERACT process. J Rheumatol.

[43] Corner J, Wright D, Hopkinson J, Gunaratnam Y, McDonald JW, Foster C (2007). The research priorities of patients attending UK cancer treatment centres: findings from a modified nominal group study. Br J Cancer.

[44] Howell M, Tong A, Wong G, Craig JC, Howard K (2012). Important outcomes for kidney transplant recipients: a nominal group and qualitative study. Am J Kidney Dis.

[45] Sanderson T, Hewlett S, Richards P, Morris M, Calnan M (2012). Utilizing qualitative data from nominal groups: exploring the influences on treatment outcome prioritization with rheumatoid arthritis patients. J Health Psychol.

[46] Urquhart-Secord R, Craig JC, Hemmelgarn B, Tam-Tham H, Manns B, Howell M, Polkinghorne KR, Kerr PG, Harris DC, Thompson S, et al. Patient and caregiver priorities for outcomes in hemodialysis: an international nominal group technique stud. Am J Kidney Dis. 2016. doi: 10.1053/j.ajkd.2016.02.037. [Epub ahead of print]. 10.1053/j.ajkd.2016.02.037 26968042

[47] Jones J, Hunter D (1995). Qualitative research: consensus methods for medical and health services research. BMJ.

[48] MacPhail A (2001). Nominal group technique: a useful method for working with young people. Br Educ Res J.

[49] Tunnicliffe DJ, Singh-Grewal D, Chaitow J, Mackie F, Manolios N, Lin MW, O’Neill SG, Ralph AF, Craig JC, Tong A (2016). Lupus means sacrifices – the perspectives of adolescents and young adults with systemic lupus erythematosus. Arthritis Care Res.

[50] Ronen GM, Rosenbaum P, Law M, Streiner DL (2001). Health-related quality of life in childhood disorders: a modified focus group technique to involve children. Qual Life Res.

[51] Tong A, Sainsbury P, Craig JC (2007). Consolidated criteria for reporting qualitative research (COREQ): a 32 item checklist for interviews and focus groups. Int J Qual Health Care.

[52] Blackwood B, Ringrow S, Clarke M, Marshall J, Rose L, Williamson P, McAuley D (2015). Core Outcomes in Ventilation Trials (COVenT): protocol for a core outcome set using a Delphi survey with a nested randomised trial and observational cohort study. Trials.

[53] Chiarotto A, Terwee CB, Deyo RA, Boers M, Lin CW, Buchbinder R, Corbin TP, Costa LO, Foster NE, Grotle M (2014). A core outcome set for clinical trials on non-specific low back pain: study protocol for the development of a core domain set. Trials.

[54] MacLennan S, Bekema HJ, Williamson PR, Campbell MK, Stewart F, MacLennan SJ, N’Dow JM, Lam TB (2015). A core outcome set for localised prostate cancer effectiveness trials: protocol for a systematic review of the literature and stakeholder involvement through interviews and a Delphi survey. Trials.

[55] Chiarotto A, Deyo RA, Terwee CB, Boers M, Buchbinder R, Corbin TP, Costa LO, Foster NE, Grotle M, Koes BW (2015). Core outcome domains for clinical trials in non-specific low back pain. Eur Spine J.

[56] van’t Hooft J, Duffy JM, Daly M, Williamson PR, Meher S, Thom E, Saade GR, Alfirevic Z, Mol BW, Khan KS (2016). A core outcome set for evaluation of interventions to prevent preterm birth. Obstet Gynecol.

[57] Potter S, Holcombe C, Ward JA, Blazeby JM (2015). Development of a core outcome set for research and audit studies in reconstructive breast surgery. Br J Surg.

[58] Sinha IP, Smyth RL, Williamson PR (2011). Using the Delphi technique to determine which outcomes to measure in clinical trials: recommendations for the future based on a systematic review of existing studies. PLoS Med.

[59] Linstone HA, Turoff M (1975). The Delphi Method: techniques and applications.

[60] Atkins R, Tolson H, Cole BR (2005). Stability of response characteristics of a Delphi panel: application of bootstrap data expansion. BMC Med Res Methodol.

[61] Schunemann H, Brozek J, Oxman AD (2009). GRADE handbook for grading quality of evidence and strength of recommendation, vol. version 3.2.

